# Flavonoids and a New Polyacetylene from *Bidens parviflora* Willd

**DOI:** 10.3390/molecules13081931

**Published:** 2008-08-28

**Authors:** Yu-Lan Li, Jun Li, Nai-Li Wang, Xin-Sheng Yao

**Affiliations:** 1 Department of Natural Products Chemistry, Shenyang Pharmaceutical University, 103 Wenhua Road, Shenhe District, Shenyang, Liaoning 110016, P.R. China; E-mail: liyulan@szda.gov.cn (Yu-Lan Li); 2 Institute for Drug Control, 1024, Beihuan Road, Futian District, Shenzhen, Guangdong 518029, P.R. China; E-mail: lijun@szda.gov.cn (Jun Li)

**Keywords:** Keywords*Bidens* parviflora Willd, Polyacetylene, Flavonoids

## Abstract

Abstract Fifteen flavonoids, **1**-**7 **and**9**-**16**, and a polyacetylene, **8**, were isolated from the ethanol extract of the dried whole plant of *Bidens parviflora* Willd. by various chromatographic techniques. Their structures have been elucidated on the basis of spectroscopic analyses and chemical studies. Compound **8** is new and was identified as 3-(*R*),8(*E*)-decene-4,6-diyne-1,3,10-triol. All the flavonoid compounds were isolated for the first time from this plant species.

## Introduction

**Figure 1:**
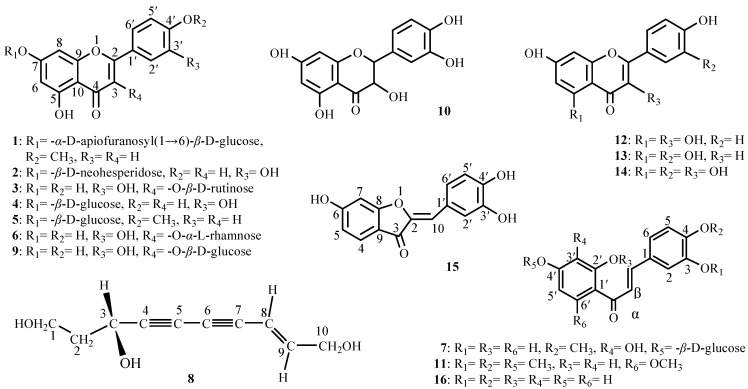
The structures of compounds **1**-**16**.

The plant *Bidens parviflora* Willd. is used in Chinese folk medicine as an antipyretic, anti-inflammatory and antirheumatic [1,2]. Flavones [3], flavonones [4], flavonoid glycosides [3,5], flavonol glycosides [6,7], chalcones [8,9], aurones [7,8], sterols [10], polyacetylene glucosides [4,9] and monoterpenes [4,10] have all been previously reported in this species. In our previous studies, sucrose esters [11], phenolic acids [12], polyacetylene glucosides [13], monoterpene glycosides [14], neolignan glucosides [15], phenolic glucosides [16] and caffeoylquinic acid derivatives [17] were isolated from this plant. As a part of an ongoing research program, this paper describes the isolation and structural determination of a new ployacetylene, **8**, and 15 known flavonoids from *B. parviflora* Willd. (Figure 1).

## Results and Discussion

Compounds **1**-**7** and **9**-**16** were identified as acacetin 7-*O*-(*α*-D-apio-furanosyl)(1→6)-*β*-D-glucoside (**1**), luteolin 7-*O*-*β*-D-neohesperidoside (**2**), quercetin 3-*O*-*β*-D-rutinoside (**3**), luteolin 7-*O*-*β*-D-glucoside (**4**), acacetin 7-*O*-*β*-D-glucoside (**5**), quercitrin (**6**), 4-methoxyl-3,2′,3′-trihydroxy-chalcone 4′-*O*-*β*-D-glucoside (**7**), quercetin 3-*O*-*β*-D-glucoside (**9**), taxifolin (**10**), 2′-hydroxy-3,4,4′,6′-tetramethoxychalcone (**11**), kaempferol (**12**), luteolin (**13**), quercetin (**14**), sulfuretin (**15**) and 3,4,2′,4′-tetrahydroxychalcone (**16**), respectively, by spectroscopic analysis (^1^H-NMR, ^13^C-NMR, UV, IR and MS) and comparisons with literature data. All these compounds have been isolated from this plant for the first time.

**Figure 2:**
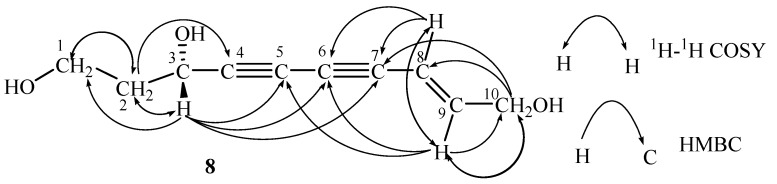
The key HMBC and ^1^H-^1^H COSY correlations of compound **8**.

Compound **8** was obtained as a brown powder with optical rotation 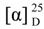: –16.9° (MeOH, *c* = 0.1), and its molecular formula was determined to be C_10_H_12_O_3_, as indicated by the [M+Na]^+^ ion at *m/z* 203.0679 (calcd. for 203.0687 [M+Na]^+^) in the HRESIMS spectrum. In the IR spectrum, absorption bands attributable to acetylene (2341 cm^-1^, 2360 cm^-1^), hydroxyl (3182 cm^-1^) and ethylene (1627cm^-1^) groups were observed. The UV spectrum of **8** was typical for an ene-diyne chromophore (λ_max _= 228, 240, 252, 266, 282 nm) [18]. The ^13^C-NMR (Table 1) and DEPT spectra of **8** present 10 carbon signals, including three methylene groups at *δ* 41.4, 59.1 and 62.6, one methine group at *δ* 60.4, two olefinic carbons at *δ* 108.6 and 148.2, and four quaternary carbons at *δ* 69.5, 74.1, 77.5 and 84.2 which were confirmed to be ethynyl carbons. Extensive analysis of the ^1^H-NMR spectrum, together with ^1^H-^1^H COSY and HMQC spectra, presented a methylene proton at *δ* 4.13 (2H, dd, *J* = 2.1, 4.7 Hz, H-10), coupled with two *E*-configured olefinic protons at *δ* 6.39 (1H, td, *J* = 4.7, 15.9 Hz, H-8) and *δ* 5.79 (1H, dtd, *J* = 0.7, 2.1, 15.9 Hz, H-9) indicating a methylene allyl moiety, two methylene protons at *δ* 3.69 (2H, m, H-1) and *δ* 1.88 (2H, m, *J* = 6.8 Hz, H-2), one methine proton at *δ* 4.57 (1H, t, *J* = 6.8 Hz, H-3). In the COSY spectrum, the correlations between *δ* 3.69 (H-1) and *δ* 1.88 (H-2), *δ* 1.88 (H-2) and *δ* 4.57 (H-3) suggested the presence of a CH_2_CH_2_CH moiety. In the HMBC spectrum, heteronuclear multiple-bond connectivity between the following: *δ*_H_ 5.79 (H-9)/*δ*_C_ 77.5, *δ*_H_ 6.39 (H-8)/*δ*_C_ 77.5 and *δ*_C_ 74.1, *δ*_H_ 4.13 (H-10)/*δ*_C_ 77.5 (C-7) could be observed; furthermore, the intensity of correlations between *δ*_H_ 6.39/*δ*_C_ 77.5 was weaker than that between *δ*_H_ 6.39/*δ*_C_ 74.1, suggesting that *δ*_C_ 74.1 and *δ*_C_ 77.5 form a alkynyl group and *δ*_C_ 77.5 directly connected with *δ*_C_ (148.2) of the CH=CHCH2 moiety, while *δ*_C_ 69.5 and *δ*_C_ 84.7 form another alkynyl. The peak at *δ*_H_ 4.57 (H-3) correlates simultaneously with *δ*_C_ 84.7, 69.5, 77.5 and 74.1, and together with *δ*_H_ 5.37 (H-9) presents a correlation with *δ*_C_ 69.5, suggesting two adjacent alkynyls, and *δ*_C_ 60.4 of the CH_2_CH_2_CH moiety is connected to *δ*_C_ 84.7. Thus, based on the chemical shifts of protons and carbons, the planar structure of compound **8** was determined to be 8-(*E*)-decene-4,6-diyne-1,3,10-triol. All ^1^H- and ^13^C-NMR signals as shown in Table 1 were assigned according to DEPT, HMQC, HMBC and ^1^H-^1^H COSY experiments. Figure 2 shows the key correlations presented in the ^1^H-^1^H COSY and HMBC spectra of **8**. 

**Table 1:** ^13^C-NMR (100 MHz, in CD_3_OD) and ^1^H-NMR (400 MHz) data of compound **8**.

Position	*δ*_C_ (ppm)	*δ*_H_ (ppm)	HMBC (H to C)
1	59.1	3.69 (2H, m)	C-2, 3
2	41.4	1.88 (2H, m)	C-1, 3, 4
3	60.4	4.57 (1H, t, *J* = 6.8 Hz)	C-1, 2, 4, 5, 6, 7
4	84.2		
5	69.5		
6	74.1		
7	77.5		
8	148.2	6.39 (1H, td, *J* = 4.7, 15.9 Hz )	C-6, 7
9	108.6	5.79 (1H, dtd, *J* = 0.7, 2.1, 15.9 Hz)	C-5, 6, 8, 9
10	62.6	4.13 (2H, dd, *J* = 2.1, 4.7 Hz )	C-7, 8, 9

**Figure 3:**
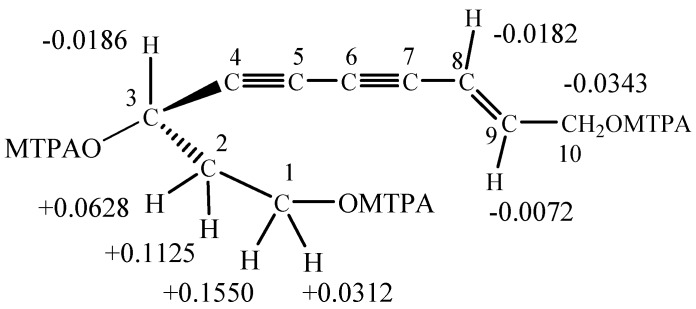
*δ* = *δ_S_–δ_R_* values (ppm) obtained from the MTPA ester of **8** in CDCl_3_ at 25 °C.

The stereochemistry at the chiral center (C-3) in compound **8** was determined using the modified Mosher method on the *R*-(+)-*α*-methoxy-*α*-trifluoromethylphenylacytyl (MTPA) and *S*-(-)-MTPA esters of **8**. In the ^1^H-NMR spectrum of the *R*-(+)-MTPA ester, the H-1 and H-2 protons appeared upfield, suggesting an effect of the MTPA pheyl ring [19]. In contrast, H-8, H-9 and H-10 were downfield from the corresponding *S*-(-)-MTPA ester. The result was shown in Figure 3. Thus, the absolute configuration at C-3 of **8** was determined to be *R*.

## Experimental

### General

UV spectra were measured in MeOH using a Shimadzu UV2401PC spectrophotometer (Shimadzu Co., Japan) and optical rotations were determined on a JASCO P-1020 polarimeter in MeOH. IR spectra were recorded on a Shimadau FTIR8400 spectrophotometer (Shimadzu Co., Japan) using KBr discs as stated. ESI-MS were obtained using a Bruker Esquire 2000 mass spectrometer (Bruker Co., Germany) and HR-ESI-MS were recorded on a Micromass Q-TOF mass spectrometer. NMR spectra were recorded on a Bruker AVANCE 400 NMR spectrometer (^1^H at 400 MHz, ^13^C at 100 MHz, Bruker Co., Germany). Chemical shifts (*δ*) are shown in ppm relative to TMS as internal standard and coupling constants (*J*) are given in Hz. The solvent used was DMSO-d_6_, unless otherwise stated. Preparative HPLC was carried out on a Shimadzu Pak equipped with a UV-Vis detector (Shimadzu Co., Japan) using a Shim-pack PREP-ODS column (5 μ, 10×250 mm, Shimadzu Co., Japan). Open column chromatography was carried out on D101 macroporous adsorption resin (Tianjin Nankai Daxue Chemical Plant, P. R. China), silica gel H60 (200-300 mesh, Qingdao Haiyang Chemical Group Co., P. R. China), Sephadex LH-20 (Amersham Pharmacia Biotech Co., UK) and ODS (60-80 μm, Merck Co., Germany) as packing materials. Thin-layer chromatography was performed on silica gel H60 plates (Qingdao Haiyang Chemical Group Co., P.R. China) and RP-18 plate (Merck Co., Germany). 

### Plant material

The whole plant material of *Bidens parviflora* Willd. was collected in July 2003 in Liaoning province, P.R. China, and was identified by Prof. Weichun Wu (Department of Medical Plants, Shenyang Pharmaceutical University, Shenyang, P.R. China). A voucher specimen (99-DHS-953) was deposited in the herbarium of the Department of Natural Products Chemistry, Shenyang Pharmaceutical University, Shenyang, P. R. China.

### Extraction and Isolation

The dried whole plant of *B. parviflora* Willd. (6.0 kg) was extracted three times with 60% EtOH (48 L) under reflux. The resulting EtOH extract (704 g) was dissolved in water (1.0 L) and applied to a D101 macroporous adsorption resin column, then eluted with water, 30% EtOH, 50% EtOH and 95% EtOH, respectively, to yield four fractions. The 30% EtOH eluate (BPB, 72 g) was subjected to silica gel column chromatography using a gradient solvent system (CHCl_3_-MeOH = 100: 0→ 98: 2→ 95: 5→ 9: 1→ 8: 2→ 7: 3→ 6: 4→ 0: 1) to give twelve fractions BPB-1 to BPB-12. The BPB-3 fraction (5.45 g) was subjected to ODS column chromatography, eluted with gradient of increasing MeOH (MeOH-H_2_O = 20: 80→ 40: 60→ 60: 40→ 90: 10), to give six fractions BPB-3a to BPB-3e; BPB-3c (940 mg) was purified by silica gel chromatography (gradient elution separation with a CHCl_3_-MeOH system: 95: 5→ 9: 1→ 85: 15→ 8: 2), followed by preparative HPLC (ODS, MeOH-H_2_O = 48:52) to yield compounds **1** (10 mg) and **2** (13 mg). BPB-3d (1.85 g) was subjected to Sephadex LH-20 column chromatography (MeOH-H_2_O = 50:50), followed by preparative HPLC (ODS, MeOH-H_2_O = 55:45) to obtained **3** (68 mg) and **4** (25 mg). The BPB-6 fraction (7.24 g) was subjected to ODS column chromatography, eluted with an increasing gradient of MeOH (MeOH-H_2_O = 10:90→ 30:70→ 50:50→ 70:30) to give six fractions BPB-6a to BPB-6e. BPB-6b (2.40 g) and BPB-6c (2.02 g) were subjected to Sephadex LH-20 column chromatography (MeOH- H_2_O = 1:1), followed by ODS column chromatography and preparative HPLC (ODS, MeOH-H_2_O = 35: 65) to yield **5** (12 mg), **6** (45 mg), **7** (13 mg), **8** (30 mg) and **9** (22 mg). 

The 60% EtOH eluate (BPC, 48 g) was subjected to silica gel column chromatography using a gradient solvent system (hexane-acetone =100: 0→ 98: 2→ 95: 5→ 9: 1→ 8: 2→ 7: 3→ 6: 4→ 0: 1) to give twelve fractions BPC-1 to BPC-12. Fraction BPC-1 (3.34 g) was subjected to silica gel column chromatography, eluted with hexane and ethyl acetate in increasing order of polarity, to give eight fractions BPC-1a to BPC-1h. BPC-1g (107 mg) was purified with Sephadex LH-20 column chromatography (CHCl_3_-MeOH = 1:1) to yield **10** (23 mg). BPC-3 (5.43 g) and BPC-6 (3.06 g) were subjected to Sephadex LH-20 column chromatography (CHCl_3_-MeOH = 1:1) followed by silica gel column chromatography (gradient elution separation with CHCl_3_-acetone system) to obtain **11** (30 mg), **12** (24 mg), **13** (15 mg), **14** (20 mg), **15** (16 mg) and **16** (10 mg).

*Acacetin 7-O-(α-D-apio-furanosyl) (1→6)-β-D-glucoside* (**1**) [20]: C_27_H_30_O_14_; yellow needle (MeOH); mp 244-245 °C; [α]_D_ 23.0° (MeOH, *c* = 0.23, 24 °C); ESI-MS (positive) *m/z* 579 [M+H]^+^; UV (MeOH) λ_max_ nm (logε): 268 (4.34), 325 (4.40); IR *ν*_max_ (KBr): 3424, 2927, 1652, 1610, 1500, 1432, 1303, 1257, 1174, 1058, 831 cm^-1^; ^1^H-NMR *δ*: 8.06 (2H, d, *J* = 9.1 Hz, H-2′, 6'), 7.13 (2H, d, *J* = 9.1 Hz, H-3′, 5′), 6.95 (1H, s, H-3), 6.46 (1H, d, *J* = 2.3 Hz, H-6), 6.82 (1H, d, *J* = 2.3 Hz, H-8), , 3.87 (3H, s, 4′-OCH_3_), 5.07 (1H, d, *J* = 7.5 Hz, glc-H-1), 4.82 (1H, d, *J* = 3.2 Hz, api-H-1); ^13^C-NMR *δ*: 163.8 (s, C-2), 103.8 (d, C-3), 182.0 (s, C-4), 161.0 (s, C-5), 99.6 (d, C-6), 162.9 (s, C-7), 94.8 (d, C-8), 156.9 (s, C-9), 105.4 (s, C-10), 122.7 (s, C-1′), 114.6 (d, C-2′, 6′), 128.4 (d, C-3′, 5′), 162.4 (s, C-4′), 55.6 (q, 4′-OCH_3_), 99.8 (d, glc-C-1), 72.9 (d, glc-C-2), 76.2 (d, glc-C-3), 69.6 (d, glc-C-4), 75.5 (d, glc-C-5), 67.3 (t, glc-C-6), 109.1 (d, api-C-1), 75.9 (d, api-C-2), 78.7 (s, api-C-3), 73.3 (t, api-C-4), 63.3 (t, api-C-5). Resonance assignments were based on ^1^H-^1^H COSY, HMQC and HMBC spectra.

*Luteolin 7-O-β-D-neohesperidoside* (**2**) [21,22]: C_28_H_32_O_15_; yellow needles; mp 249-251 °C; ESI-MS (positive) *m/z* 609 [M+H]^+^;^ 1^H-NMR *δ*: 7.44 (1H, dd, *J* = 8.3, 2.1 Hz, H-6′), 7.40 (1H, d, *J* = 2.1 Hz, H-2′), 6.90 (1H, *J* = 8.3 Hz, H-5′), 6.75 (1H, s, H-3), 6.74 (1H, d, *J* = 2.1 Hz, H-8), 6.38 (1H, d, *J* = 2.1 Hz, H-6), 5.25 (1H, d, *J* = 7.3 Hz, glc-H-1), 5.14 (1H, d, *J* = 1.2 Hz, rha-H-1), 1.20 (3H, d, *J* = 6.2 Hz, rha-CH_3_); ^13^C-NMR *δ*: 166.3 (s, C-2), 105.1 (d, C-3), 183.7 (s, C-4), 163.0 (s, C-5), 99.6 (d, C-6), 164.4 (s, C-7), 92.6 (d, C-8), 158.8 (s, C-9), 107.3 (s, C-10), 123.2 (s, C-1′), 115.4 (d, C-2′), 147.6 (s, C-3′),151.8 (s, C-4′), 117.9 (d, C-5′), 121.0 (d, C-6′), 102.3 (d, glc-C-1), 101.2 (d, rha-C-1), 78.8 (d, glc-C-2), 79.0 (d, glc-C-3), 72.2 (d, glc-C-4), 78.2 (d, glc-C-5), 62.4 (d, glc-C-6), 72.4 (d, rha-C-2), 71.5 (d, rha-C-3), 73.7 (d, rha-C-4), 70.2 (d, rha-C-5), 19.9 (q, rha-CH_3_). 

*Quercetin 3-O-β-D-rutinoside* (**3**) [23]: C_27_H_30_O_16_; yellow powder; [α]26 D -2.8° (*c* = 0.1, MeOH); ESI-MS^n^ (positive and negative) *m/z* 633 [M+Na]^+^, 487[M+Na-146]^+^, 609[M-H]^-^, 301[M-H-(146+162)]^-^; UV (MeOH) λ_max_ nm (logε): 355 (4.09), 256 (4.20);^ 1^H-NMR *δ*: 6.21 (1H, d, *J* = 2.2 Hz, H-6), 6.40 (1H, d, *J* = 2.2 Hz, H-8), 7.67 (1H, d, *J* = 2.5 Hz, H-2′), 6.87 (H, d, *J* = 8.3 Hz, H- 5′), 7.63 (1H, dd, *J* = 8.6, 2.2 Hz, H-6′), 5.11(1H, d, *J* = 7.7 Hz, glc-H-1) 4.52 (1H, d, *J* = 1.5 Hz, rha-H-1), 1.12 (3H, d, *J* = 6.1 Hz, rha-H-6); ^13^C-NMR *δ*: 159.4 (C-2), 135.7 (s, C-3), 179.5 (s, C-4), 163.0 (s, C-5), 99.9 (d, C-6), 166.1 (s, C-7), 94.9 (d, C-8), 158.6 (s, C-9), 105.7 (s, C-10), 123.2 (s, C-1′), 116.1 (d, C-2′), 145.9 (s, C-3′), 149.9 (s, C-4′), 117.7 (d, C-5′), 123.6 (d, C-6′), 104.8 (d, glc-C-1), 75.8 (d, glc-C-2), 78.2 (d, glc-C-3), 71.4 (d, glc-C-4), 77.3 (d, glc-C-5), 68.6 (t, glc-C-6), 102.5 (d, rha-C-1), 72.3 (d, rha-C-2), 72.1 (d, rha-C-3), 74.0 (d, rha-C-4), 69.8 (d, rha-C-5), 17.9 (q, rha-C-6).

*Luteolin 7-O-β-D-glucoside* (**4**) [24]: C_21_H_20_O_11_; yellow needles; mp 257-259 °C; ESI-MS (positive) *m/z* 449 [M+H]^+^; ^1^H-NMR (DMSO-*d*_6_) *δ*: 13.0 (1H, s, br, 5-OH), 6.74 (1H, s, H-3), 6.44 (1H, d, *J* = 2.1 Hz, H-6), 6.78 (1H, d, *J* = 2.1 Hz, H-8), 7.42 (1H, d, *J* = 2.2 Hz, H-2′), 6.90 (1H, d, *J* = 8.2 Hz, H-5′), 7.44 (1H, dd, *J* = 8.2, 2.2 Hz, H-6′), 5.08 (1H, d, *J* = 7.3 Hz, glc-H-1); ^13^C-NMR (DMSO-*d*_6_) *δ*: 163.7 (s, C-2), 102.4 (d, C-3), 181.1 (s, C-4), 160.3 (s, C-5), 98.8 (d, C-6), 162.1 (s, C-7), 93.9 (d, C-8), 156.2 (s, C-9), 104.5 (s, C-10), 120.6 (s, C-1′), 112.8 (d, C-2′), 145.0 (s, C-3′), 149.1 (s, C-4′), 115.2 (d, H-5′), 118.4 (d, H-6′), 99.1 (d, glc-C-1), 72.3 (d, glc-C-2), 76.4 (d, glc-C-3), 68.8 (d, glc-C-4), 75.6 (d, glc-C-5), 59.8 (t, glc-C-6).

*Acacetin 7-O-β-D-glucoside* (**5**) [25]: C_22_H_22_O_10_; yellow powder; ESI-MS (positive) *m/z* 447 [M+H]^+^; ^1^H-NMR *δ*: 12.92 (1H, s, 5-OH), 6.96 (1H, s, H-3), 6.46 (1H, d, *J* = 2.2 Hz, H-6), 6.86 (1H, d, *J* = 2.2 Hz, H-8), 8.07 (2H, d, *J* = 9.0 Hz, H-2′, H-6′), 7.14 (2H, d, *J* = 9.0 Hz, H-3′, H-5′), 5.07 (1H, d, *J* = 7.7 Hz, glc-H-1), 3.89 (3H, s, 4′-OCH_3_); ^13^C-NMR *δ*: 163.8 (s, C-2), 103.7 (d, C-3) , 181.7 (s, C-4), 161.0 (s, C-5), 99.5 (d, C-6), 162.9 (s, C-7), 94.8 (d, C-8), 156.8 (s, C-9), 105.3 (s, C-10), 122.6 (s, C-1′), 128.4 (d, C-2′), 114.6 (s, C-3′), 162.4 (s, C-4′), 114.6 (d, H-5'), 128.4 (d, H-6'), 99.8 (d, glc-C-1), 72.9 (d, glc-C-2), 77.1 (d, glc-C-3), 69.5 (d, glc-C-4), 76.3 (d, glc-C-5), 60.5 (t, glc-C-6), 55.5 (q, 4′-OCH_3_). 

*Quercitrin* (**6**) [26]: C_21_H_20_O_11_; yellow powder; ESI-MS (positive) *m/z* 449 [M+H]^+^; ^1^H-NMR *δ*: 12.65 (1H, s, 5-OH), 6.86 (1H, d, *J* = 8.3 Hz, H-5′), 6.39 (1H, d, *J* = 2.2 Hz, H-8), 7.30 (1H, d, *J* = 2.1 Hz, H-2′), 7.25 (1H, dd, *J* = 2.1, 8.3 Hz, H-6′), 6.20 (1H, d, *J* = 2.2 Hz, H-6), 5.25 (1H, d, *J* = 1.4 Hz, rha-H-1), 3.97 (1H, t, *J* = 1,4 Hz, rha-H-2), 0.81 (3H, d, *J* = 6.0 Hz, rha-CH_3_); ^13^C-NMR *δ*: 157.2 (s, C-2), 134.2 (s, C-3), 177.7 (s, C-4), 161.3 (s, C-5), 98.7 (d, C-6), 164.2 (s, C-7), 93.6 (d, C-8), 156.4 (s, C-9), 104.0 (s, C-10), 120.7 (s, C-1′), 115,4 (d, C-2′), 145.2 (s, C-3′), 148.4 (s, C-4′), 115.6 (d, C-5′), 121.1 (d, C-6′), 101.8 (d, rha-C-1), 70.5 (d, rha-C-2), 70.3 (d, rha-C-3), 71.2 (d, rha-C-4), 70.0 (d, rha-C-5), 17.4 (q, rha-CH_3_). 

*4-Methoxy-3,2′,3′-trihydroxychalcone 4′-O-β-D-glucoside* (**7**) [27,28]: C_22_H_24_O_11_; yellow powder; ESI-MS (positive) *m/z* 465 [M+H]^+^, 302 [M-162]^+^; UV (MeOH) λ_max_ nm (logε): 229 (3.65), 312 (3.22); IR *ν*_max_ (KBr) cm^-1^: 3367, 2925, 1637, 1567, 1511, 1448, 1367, 1272, 1087; ^1^H-NMR (CD_3_OD) *δ*: 7.79 (1H, d, *J* = 15.4Hz, *β*-H), 7.62 (1H, d, *J* = 15.4 Hz, *α*-H), 7.25 (1H, d, *J* = 2.2 Hz, H-2), 7.00 (1H, d, *J* = 8.3 Hz, H-5), 7.22 (1H, dd, *J* = 8.3, 2.2Hz, H-6), 7.65 (1H, d, *J* = 9.2Hz, H-6′), 6.86 (1H, d, *J* = 9.2 Hz, H-5′), 3.91(3H, s, 4-OCH_3_), 4.98 (H, d, *J* = 7.3 Hz, glc-H-1); ^13^C-NMR (CD_3_OD) *δ*: 119.3 (d, C-*α*), 146.5 (d, C-*β*), 194.6 (s, C=O), 129.4 (s, C-1), 115.3 (d, C-2), 152.0 (s, C-3), 148.1 (s, C-4), 119.3 (d, C-5), 123.8 (d, C-6), 117.4 (s, C-1′), 146.5 (s, C-2′), 143.3 (s, C-3′), 165.1 (s, C-4′), 108.2 (d, H-5′), 122.7 (d, H-6′), 102.7 (d, glc-C-1), 74.8 (d, glc-C-2), 78.5 (d, glc-C-3), 71.3 (d, glc-C-4), 77.6 (d, glc-C-5), 62.5 (t, glc-C-6), 56.5 (q, 4′-OCH_3_). 

*Quercetin 3-O-β-D-glucoside* (**9**) [29]: C_21_H_20_O_12_; yellow powder; ESI-MS (positive and negative) *m/z* 487 [M+Na]^+^ , 325 [M+Na-162]^+^, 463 [M-H]^-^, 301 [M-H-162]^-^; UV (MeOH) λ_max_ nm (logε): 356 (4.05), 297 (3.83), 256 (4.13); ^1^H-NMR *δ*: 12.62 (1H, s, 5-OH), 10.85 (1H, s, br, 7-OH), 9.72 (1H, s, br, 4′-OH), 9.17 (1H, s, br, 3′-OH), 6.20 (1H, d, *J* = 2.0 Hz, H-6), 6.41 (1H, d, *J* = 2.0 Hz, H-8), 7.53 (1H, d, *J* = 1.9 Hz, H-2′), 6.82 (1H, d, *J* = 8.3 Hz, H-5′), 7.66 (1H, dd, *J* = 8.3, 1.9 Hz, H-6′), 5.37 (1H, d, *J* = 7.6 Hz, glc-H-1); ^13^C-NMR *δ*: 156.2 (s, C-2), 133.4 (s, C-3), 177.4 (s, C-4), 161.2 (s, C-5), 98.6 (d, C-6), 164.1 (s, C-7), 93.4 (d, C-8), 156.2 (s, C-9), 103.9 (s, C-10), 121.0 (s, C-1′), 115.1 (d, C-2′), 144.8 (s, C-3′), 148.4 (s, C-4′), 115.9 (d, C-5′), 121.9 (d, C-6′), 101.8 (d, glc-C-1), 74.0 (d, glc-C-2), 77.5 (d, glc-C-3), 69.8 (d, glc-C-4), 76.4 (d, glc-C-5), 60.8 (t, glc-C-6). 

*Taxifolin* (**10**) [30]: C_15_H_12_O_7_; white powder; ESI-MS (positive) *m/z* 327 [M+Na]^+^; ^1^H-NMR *δ*: 11.87 (1H, s, 5-OH), 10.79 (1H, s, 7-OH), 8.99 (1H, s, 4′-OH), 8.94 (1H, s, 3′-OH), 4.96 (1H, d, *J* = 11.2 Hz, H-2), 4.48 (1H, d, *J* = 11.2 Hz, H-3), 5.84 (1H, d, *J* = 2.1 Hz, H-6), 5.89 (1H, d, *J* = 2.1 Hz, H-8), 6.85 (1H, s, br, H-2′) , 6.71 (1H, d, *J* = 8.0 Hz, H-5′), 6.73 (1H, d, *J* = 8.0 Hz, H-6′); ^13^C-NMR *δ*: 83.5 (d, C-2), 72.0 (d, C-3), 198.2 (s, C-4), 163.8 (s, C-5), 96.4 (d, C-6), 167.2 (s, C-7), 95.4 (d, C-8), 163.0 (s, C-9), 101.0 (s, C-10), 128.5 (s, C-1′), 115.6 (d, C-2′), 145.4 (s, C-3′), 146.2 (s, C-4′), 115.8 (d, C-5′), 119.8 (d, C-6′).

*2′-Hydroxy-3,4,4′,6′-tetramethoxychalcone* (**11**) [31]: C_19_H_20_O_6_; yellow powder; ESI-MS (positive and negative) *m/z* 345 [M+H]^+^, 343 [M-H]^-^; ^1^H-NMR (acetone-*d_6_*) *δ*: 7.91 (1H, d, *J* = 15.4 Hz, H-*α*), 7.75 (1H, d, *J* = 15.4 Hz, H-*β*), 7.33 (1H, d, *J* = 2.0 Hz, H-2), 7.03 (1H, d, *J* = 8.2 Hz, H-5), 7.30 (1H, dd, *J* = 8.2, 2.0 Hz, H-6), 6.13 (1H, d, *J* = 2.4 Hz, H-3′), 6.09 (1H, d, *J* = 2.4 Hz, H-5′), 4.01 (3H, s, 4′-OCH_3_), 3.91 (3H, s, 3-OCH_3_), 3.88 (6H, 4, 6′-OCH_3_); ^13^C-NMR (acetone-*d_6_*) *δ*: 193.4 (s, C=O), 126.0 (d, C-*α*), 143.6 (d, C-*β*), 129.3 (s, C-1), 111.7 (d, C-2), 150.6 (s, C-3), 152.7 (s, C-4), 112.6 (d, C-5), 123.7 (d, C-6), 106.9 (s, C-1′), 169.1 (s, C-2′), 91.8 (d, C-3′), 163.7 (s, C-4′), 94.7 (d, C-5′), 167.4 (s, C-6′), 56.2 (q, 3-OCH_3_), 56.1 (q, 4-OCH_3_), 56.5 (q, 4′-OCH_3_), 56.1 (q, 6′-OCH_3_). 

*Kaempferol* (**12**) [32]: C_15_H_10_O_6_; yellow powder; ESI-MS (negative) *m/z* 285 [M-H]^-^; UV (MeOH) λ_max_ nm (logε): 254 (4.15), 366 (4.02); ^1^H-NMR (acetone-*d*_6_) *δ*: 6.27 (1H, d, *J* = 2.2 Hz, H-6), 6.54 (1H, d, *J* = 2.2 Hz, H-8), 8.14 (2H, d, *J* = 8.0 Hz, H-2′, 6′), 7.02 (2H, d, *J* = 8.0 Hz, H-3′, 5′); ^13^C-NMR (acetone-*d*_6_) 147.1 (s, C-2), 136.7 (s, C-3), 176.6 (s, C-4), 162.4 (s, C-5), 99.2 (d, C-6), 165.0 (s, C-7), 94.5 (d, C-8), 157.8 (s, C-9), 104.2 (s, C-10), 123.4 (s, C-1′), 130.5 (d, C-2′, 6′), 116.4 (d, C-3′, 5′), 160.2 (s, C-4′).

*Luteolin* (**13**) [25]: C_15_H_10_O_6_; yellow needles; mp 328-330 °C; ESI-MS (positive) *m/z* 287 [M+H]^+^; ^1^H-NMR *δ*: 6.74 (1H, s, H-3), 6.44 (1H, d, *J* = 1.9 Hz, H-6), 6.79 (1H, d, *J* = 1.9 Hz, H-8), 7.42 (1H, d, *J* = 2.2, H-2′), 6.90 (1H, d, *J* = 8.2, H-5′), 7.45 (1H, dd, *J* = 8.2, 2.2, H-6′); ^13^C-NMR *δ*: 164.5 (s, C-2), 103.1 (d, C-3), 181.9 (s, C-4), 161.1 (s, C-5), 99.1 (d, C-6), 163.9 (s, C-7), 94.2 (d, C-8), 156.9 (s, C-9), 104.3 (s, C-10), 121.3 (s, C-1′), 113.5 (d, C-2′), 145.8 (s, C-3′), 150.3 (s, C-4′), 115.9 (d, C-5′), 119.2 (d, C-6′).

*Quercetin* (**14**) [25,33]: C_15_H_10_O_7_; yellow needles; mp 310-312 °C; ESI-MS (positive and negative) *m/z* 303 [M+H]^+^, 301 [M-H]^-^; UV (MeOH) λ_max_ nm (logε): 255 (4.26), 370 (4.20); ^1^H-NMR *δ*: 12.48 (1H, s, 5-OH), 10.75 (1H, s, br, 3-OH), 9.56 (1H, s, br, 7-OH), 9.32 (2H, s, br, 2×-OH), 6.19 (1H, d, *J* = 2.0 Hz, H-6), 6.41 (1H, d, *J* = 2.0 Hz, H-8), 7.68 (1H, d, *J* = 2.2 Hz, H-2′), 6.89 (1H, d, *J* = 8.5 Hz, H-5′), 7.54 (1H, dd, *J* = 2.2, 8.5 Hz, H-6′); ^13^C-NMR *δ*: 147.6 (s, C-2), 135.6 (s, C-3), 175.8 (s, C-4), 160.6 (s, C-5), 98.1 (d, C-6), 163.8 (s, C-7), 93.3 (d, C-8), 156.1 (s, C-9), 103.0 (s, C-10), 121.9 (s, C-1′), 115.0 (d, C-2′), 145.0 (s, C-3′), 146.7 (s, C-4′), 115.5 (d, C-5′), 120.0 (d, C-6′).

*Sulfuretin* (**15**) [34]: C_15_H_10_O_5_; yellow powder; ESI-MS (positive and negative) *m/z* 271 [M+H]^+^, 269.0 [M-H]^-^; ^1^H-NMR *δ*: 6.84 (1H, d, *J* = 8.3 Hz, H-4), 6.70 (1H, dd, *J* = 8.3, 2.0 Hz, H-5), 6.74 (1H, d, *J* = 2.0 Hz, H-7), 6.63 (1H, s, H-10), 7.45 (1H, d, *J* = 1.2 Hz, H-2′), 7.59 (1H, d, *J* = 8.3 Hz, H-5′), 7.24 (1H, dd, *J* = 8.3, 1.2 Hz, H-6′); ^13^C-NMR *δ*: 145.6 (s, C-2), 181.2 (s, C-3), 125.8 (d, C-4), 113.0 (d, C-5), 166.3 (s, C-6), 98.4 (d, C-7), 167.5 (s, C-8), 113.2 (s, C-9), 111.9 (d, C-10), 123.4 (s, C-1′), 118.0 (d, C-2′), 145.7 (s, C-3′), 148.0 (s, C-4′), 116.1 (d, C-5′), 124.6 (d, C-6′). 

*3,4,2′,4′-Tetrahydroxychalcone* (**16**) [35,36]: C_15_H_12_O_4_; yellowish oily substance; ESI-MS (positive and negative) *m/z* 287 [M+H]^+^, 285 [M-H]^-^; UV (MeOH) λ_max_ nm (logε): 218 (3.50), 250 (2.91), 289 (0.54); IR *ν*_max_ (KBr) cm^-1^: 2554, 1693, 1597, 1570, 1504, 1420, 1377, 1327;^ 1^H-NMR (CD_3_OD) δ: 6.70 (1H, d, *J* = 15.3 Hz, H-*α*), 6.90 (1H, d, *J* = 15.3 Hz, H-*β*), 6.37 (1H, s, H-2), 6.01 (1H, d, *J* = 8.0 Hz, H-5), 6.28 (1H, d, *J* = 8.0 Hz, H-6), 5.48 (1H, d, *J* = 2.0 Hz, H-3′), 5.67 (1H, dd, *J* = 8.9, 2.0 Hz, H-5′), 7.11 (1H, d, *J* = 8.9 Hz, H-6′); ^13^C-NMR (CD_3_OD) *δ*: 193.5 (s, C=O), 146.0 (d, C-*α*), 118.3 (d, C-*β*), 128.4 (s, C-1), 115.8 (d, C-2), 146.8 (s, C-3), 149,8 (s, C-4), 116.6 (d, C-5), 123.6 (d, C-6), 114.7 (s, C-1′), 167.4 (s, C-2′), 103.8 (d, C-3′) 166.2 (s, C-4′), 109.1 (d, C-5′), 133.2 (d, C-6′). 
